# Supplementary material for: Introgression of Heterotic Genomic Segments from Brassica carinata into Brassica juncea for Enhancing Productivity
Source: Plants (Basel). 2023 Apr 17;12(8):1677. doi: 10.3390/plants12081677 (PMC10146992; doi:10.3390/plants12081677)
Supplement: Supplementary file 1 [file plants-12-01677-s001.zip › Table S1.pdf]

Table S1 (a): Analysis of variance for seed yield and its contributing traits in a set of hybrids generated between *B. carinata* derived *B. juncea* ILs and respective genetic backgrounds along with respective parents in RCBD design

	Experiment 1a#			Coefficient of variation (%)	Experiment 1b$			Coefficient of variation (%)
Source	Replications	Treatments	Error		Replications	Treatments	Error	
d.f.	2	54	108		2	26	52	
Mean sum of squares								
Siliqua length (cm)	0.16	0.63**	0.06	6.44	0.55**	0.60**	0.09	7.57
Seeds/Siliqua	2.83*	6.02**	0.74	6.43	0.25	3.04**	0.56	5.67
Total siliqua on main shoot	187.78**	52.63**	15.77	7.99	1.25	85.72**	11.18	6.64
Total number of siliqua/plant	7600.48*	5197.65**	1806.8	13.72	7570.36*	8479.64**	1754.37	12.28
Oil content (%)	28.18**	13.38**	1.3	3.19	28.75**	6.19**	0.91	2.73
1,000-seed weight (g)	0.1	0.73**	0.14	8.06	0.07	1.58**	0.17	8.10
Harvest index (%)	9.73	22.34**	7.66	11.86	12.78	11.34**	5.26	9.67
Seed yield (t/ha)	0.1	1.37**	0.09	11.91	0.02	0.58**	0.08	11.33

# = including hybrids (D31_ILHs) generated between ILs of DRMRIJ 31 (D31_ILs) and DRMRIJ 31 along with parents

$ = including hybrids (PM30_ILHs) generated between ILs of Pusa Mustard 30 (PM30_ILs) and Pusa Mustard 30 along with parents

* significant at p = 0.1; ** significant at p = 0.05.

Table S1 (b): Analysis of variance for seed yield and its contributing traits in a set of hybrids generated between *B. carinata* derived *B. juncea* ILs and common tester SEJ 8 along with respective parents in RCBD design

	Experiment 4b#			Coefficient of variation (%)	Experiment 4c$			Coefficient of variation (%)
Source	Replications	Treatments	Error		Replications	Treatments	Error	
d.f.	2	27	54		2	13	26	
Mean sum of squares								
Siliqua length (cm)	0.001	0.48**	0.05	4.83	0.64**	0.18**	0.04	4.88
Seeds/Siliqua	2.41*	2.95**	0.54	4.93	1.69*	1.27**	0.35	3.99
Total siliqua on main shoot	6.87	40.26**	10.47	5.90	35.12*	39.14**	6.58	5.00
Total number of siliqua/plant	3615.79	5762.89**	1818.01	12.87	998.94	2612.85**	835.62	9.84
Oil content (%)	11.77*	7.78*	1.76	3.39	0.69	3.18**	0.781	2.32
1,000-seed weight (g)	0.04	0.34**	0.09	6.72	0.19	0.55**	0.07	5.62
Harvest index (%)	9.73	14.81**	5.94	11.90	17.18	7.32	6.92	10.22
Seed yield (t/ha)	0.36	0.34**	0.11	11.28	0.04	0.29**	0.09	11.26

# = including hybrids (D31_THs) generated between ILs of DRMRIJ 31 (D31_ILs) and SEJ 8 along with parents

$ = including hybrids (PM30_THs) generated between ILs of Pusa Mustard 30 (PM30_ILs) and SEJ 8 along with parents

* significant at p = 0.1; ** significant at p = 0.05.
