# Supplementary material for: Introgression of Heterotic Genomic Segments from Brassica carinata into Brassica juncea for Enhancing Productivity
Source: Plants (Basel). 2023 Apr 17;12(8):1677. doi: 10.3390/plants12081677 (PMC10146992; doi:10.3390/plants12081677)
Supplement: Supplementary file 1 [file plants-12-01677-s001.zip › Table S2.pdf]

Table S2. Mean and range for seed yield and its contributing traits among test hybrids and check hybrids

Hybrid(s)	Parameter	Traits							
		SL	SPS	SMS	TS	OC	TSW	HI	SY
DRMRIJ-31 x SEJ 8 (Check hybrid-1)	Mean	4.87	15.27	43.40	249.60	40.13	4.83	24.57	2.50
D31_ILs x SEJ 8 (Test hybrids[#])	Mean ± SD	4.78 ± 0.41	14.95 ± 1.01	55.31 ± 2.95	334.37 ± 41.57	39.22 ± 1.63	4.53 ± 0.34	26.06 ± 2.25	3.07 ± 0.33
	Range	3.90 - 5.60	13.13 - 17.13	48.00 - 61.40	272.40 - 422.80	35.67 - 41.77	3.93 - 5.20	22.47 - 30.93	2.30 - 3.57
**Critical difference (P = 0.05)**		0.37	1.20	5.29	69.79	2.17	0.49	3.98	0.56
Pusa Mustard 30 x SEJ 8 (Check hybrid-2)	Mean	4.40	13.70	47.27	269.60	38.57	5.63	23.23	2.73
PM30_ILs x SEJ 8 (Test hybrids[$])	Mean ± SD	4.38 ± 0.26	14.94 ± 0.59	51.64 ± 3.56	295.69 ± 29.85	38.24 ± 1.07	4.83 ± 0.39	25.94 ± 1.44	2.81 ± 0.33
	Range	4.00 - 4.90	13.93 - 15.73	46.00 - 57.87	230.20 - 335.40	36.43 - 39.80	4.17 - 5.43	23.43 - 27.70	2.27 - 3.23
**Critical difference (P = 0.05)**		0.35	0.99	4.30	48.51	1.48	0.46	-	0.52

SL = Siliqua length (cm); SPS = Seeds per siliqua; SMS = Total siliquae on main shoot; TS = Total siliquae/plant; OC = Oil content (%), TSW = 1000 Seed weight; HI = Harvest index (%); SY = Seed yield (t/ha)
D31_IL = *B. carinata* derived *B. juncea* introgression line in the genetic background of cultivar DRMRIJ 31
PM30_IL = *B. carinata* derived *B. juncea* introgression line in the genetic background of cultivar Pusa Mustard 30
# = Test hybrids were compared with Check hybrid-1
$ = Test hybrids were compared with Check hybrid-2
