# Supplementary material for: Introgression of Heterotic Genomic Segments from Brassica carinata into Brassica juncea for Enhancing Productivity
Source: Plants (Basel). 2023 Apr 17;12(8):1677. doi: 10.3390/plants12081677 (PMC10146992; doi:10.3390/plants12081677)
Supplement: Supplementary file 1 [file plants-12-01677-s001.zip › Table S3.pdf]

Table S3. Heterotic hybrids identified for seed yield and its contributing traits

Traits	Hybrids expressing significant Mid parent heterosis	Hybrids expressing significant standard heterosis over respective check hybrids
**SL**	D31_ILH82, D31_ILH87, D31_ILH101, D31_ILH104, D31_ILH106, D31_ILH139, D31_ILH141, D31_ILH146, D31_ILH158, PM30_ILH170, PM30_ILH177, PM30_ILH179, PM30_ILH180, PM30_ILH181, PM30_ILH182, PM30_ILH187, PM30_ILH190 **(17)**	D31_TH105, D31_TH106, D31_TH120, PM30_TH181 **(4)**
**SPS**	D31_ILH104, PM30_ILH168, PM30_ILH170, PM30_ILH180, PM30_ILH187, PM30_ILH190 **(6)**	D31_TH105, D31_TH106PM30_TH168, PM30_TH171, PM30_TH179, PM30_TH180, PM30_TH181, PM30_TH182, PM30_TH186, PM30_TH187, PM30_TH188 **(11)**
**SMS**	PM30_ILH180, PM30_ILH185, PM30_ILH186 **(3)**	D31_TH82, D31_TH87, D31_TH90, D31_TH91, D31_TH92, D31_TH94, D31_TH95, D31_TH97, D31_TH101, D31_TH104, D31_TH105, D31_TH106, D31_TH110, D31_TH115, D31_TH119, D31_TH120, D31_TH121, D31_TH128, D31_TH131, D31_TH136, D31_TH139, D31_TH140 **(22)**
**TS**	D31_ILH82, D31_ILH87, D31_ILH120, PM30_ILH179, PM30_ILH180, PM30_ILH186, PM30_ILH188 **(7)**	D31_TH87, D31_TH90, D31_TH91, D31_TH92, D31_TH94, D31_TH97, D31_TH104, D31_TH106, D31_TH115, D31_TH120, D31_TH128, D31_TH136, D31_TH139, D31_TH141, D31_TH146, D31_TH158, PM30_TH180, PM30_TH187, PM30_TH188 **(19)**
**OC**	D31_ILH87, PM30_ILH168, PM30_ILH170, PM30_ILH177, PM30_ILH179, PM30_ILH180, PM30_ILH181, PM30_ILH182, PM30_ILH185, PM30_ILH187, PM30_ILH190 **(11)**	Nil
**TSW**	D31_ILH82, D31_ILH89, D31_ILH92, D31_ILH104, D31_ILH105, D31_ILH106, D31_ILH120, D31_ILH136, D31_ILH139, D31_ILH140, D31_ILH156, D31_ILH158, PM30_ILH170, PM30_ILH177 **(14)**	Nil
**HI**	D31_ILH91, D31_ILH95, PM30_ILH179, PM30_ILH185, PM30_ILH188 **(5)**	D31_TH97, D31_TH106, D31_TH115, D31_TH120, PM30_TH182 **(5)**
**SY**	D31_ILH82, D31_ILH87, D31_ILH89, D31_ILH91, D31_ILH95, D31_ILH97, D31_ILH101, D31_ILH104, D31_ILH105, D31_ILH115, D31_ILH119, D31_ILH120, D31_ILH121, D31_ILH128, D31_ILH131, D31_ILH136, D31_ILH139, D31_ILH146, D31_ILH156, PM30_ILH168, PM30_ILH170, PM30_ILH171, PM30_ILH179. PM30_ILH180, PM30_ILH181, PM30_ILH185, PM30_ILH186, PM30_ILH187, PM30_ILH188, PM30_ILH190 **(30)**	D31_TH87, D31_TH89, D31_TH90, D31_TH91, D31_TH97, D31_TH104, D31_TH105, D31_TH106, D31_TH110, D31_TH119, D31_TH120, D31_TH121, D31_TH128, D31_TH136, D31_TH139, D31_TH140, D31_TH158 **(17)**

SL = Siliqua length (cm); SPS = Seeds per siliqua; SMS = Total siliquae on main shoot; TS = Total siliquae/plant; OC = Oil content (%), TSW = 1000 Seed weight; HI = Harvest index (%); SY = Seed yield (t/ha)
D31_ILH = Introgression line hybrid generated by hybridizing *B. carinata* derived *B. juncea* introgression line of DRMRIJ 31 and DRMRIJ 31
PM30_ILH = Introgression line hybrid generated by hybridizing *B. carinata* derived *B. juncea* introgression line of Pusa Mustard 30 and Pusa Mustard 30
D31_TH = Test hybrid generated by hybridizing *B. carinata* derived *B. juncea* introgression line of DRMRIJ 31 and SEJ 8
PM30_TH = Test hybrid generated by hybridizing *B. carinata* derived *B. juncea* introgression line of Pusa Mustard 30 and SEJ 8
