# Supplementary material for: Introgression of Heterotic Genomic Segments from Brassica carinata into Brassica juncea for Enhancing Productivity
Source: Plants (Basel). 2023 Apr 17;12(8):1677. doi: 10.3390/plants12081677 (PMC10146992; doi:10.3390/plants12081677)
Supplement: Supplementary file 1 [file plants-12-01677-s001.zip › Table S8.pdf]

Table S8. Correspondence of identified candidate genes in heterotic genomic segments transferred from *B. carinata* (BC-4) into *B. juncea* cultivars DRMRIJ 31 with already reported loci

D31_ILs	SNP ID	*A. thaliana* ortholog	Gene name	Function	Already known to influence trait(s)	References
IL89	B3 _5096242	AT1G18890	ATCDPK1	Calcium-Dependent Protein Kinase 1	Yield related traits	Aakanksha et al. [43]
IL105	B7 _30655855	AT1G73660	SIS8	Sugar Insensitive 8		
IL120	B4 _57181897	AT1G75820	ATCLV1, FLO5	Clavata 1, Flower Development 5		
IL120	B6 _7558546	AT2G01190	PDE331	Pigment Defective 331		
IL136	A9 _5340024	AT5G65920	PUB31	U-Box Protein 31		
IL136	A9 _5340056	AT3G49810	ATPUB30	U-Box Protein 30		
IL136	A9 _38524128	AT3G57990	OEP40	Outer Envelope Pore Protein 40 Chloroplastic		
IL136	B5 _2474275	AT2G36380	ATPDR6	ATP-Binding Cassette G34		
IL136	B7 _43692939	AT1G25440	BBX15	B-Box Domain Protein 15		
IL136	B7 _53707493	AT2G20580	ATRPN1A	26S Proteasome Regulatory Subunit S2 1A		
IL156	A5 _2092145	AT3G59690	IQD13	IQ-Domain 13		
IL156	A6 _37988602	AT2G01190	PDE331	Pigment Defective 331		
IL105, IL136	B3 _11420202	AT2G35940	BLH1	BEL1-Like Homeodomain 1		
IL136	B7 _55230799	AT1G19850	ARF5	Auxin Response Factor 5	Thousand seed weight (g)	Liu et al. [48]; Mathur et al. [50]
IL136	B2 _52492605	AT4G11260	SGT1B, ETA3	Enhancer Of TIR1-1 Auxin Resistance 3	Thousand seed weight (g)	Dhaka et al. [66]
IL136	B5 _1788619	AT5G13930	ATCHS, TT4	Transparent Testa 4, Chalcone Synthase		
IL136	B8 _32684991	AT4G25440	ZFWD1	Zinc Finger WD40 Repeat Protein 1	Seed yield, thousand seed weight (g), siliqua length (cm)	Aakanksha et al. [43]; Kumar et al. [52]
IL136, IL156	B8 _67714395	AT1G14687	AtHB32, ZHD14	Homeobox Protein 32, zinc finger homeodomain 14		
IL105, IL156	B5 _50104581	AT3G15030	MEE35, TCP4	TCP Family Transcription Factor 4, Maternal Effect Embryo Arrest 35	Yield related traits, Thousand seed weight (g)	Aakanksha et al. [43]; Mathur et al. [50]
IL136	B1 _21874370	AT2G40970	MYBC1	Myb Domain Protein		
IL156	A4 _22369916	AT2G45580	CYP76C3	Cytochrome P450, Family 76, Subfamily C, Polypeptide 3		
IL136	B2 _45604564	AT3G47600	MYB94	Myb Domain Protein 94		
IL156	B7 _31937306	AT1G72010	TCP22	TCP Domain Protein 22		
IL105	A5 _7736295	AT2G30470	VAL1, HSI2	High-Level Expression Of Sugar-Inducible Gene 2, Viviparous1/ABI3-LIKE1	Thousand seed weight (g)	Dhaka et al. [53]
IL105	A5 _7736298	AT4G32010	HSL1, VAL2	Viviparous1/ABI3-LIKE2, HSI2-Like 1		
IL136	B2 _36130560	AT2G13790	BKK1	BRI1- Associated Kinase 7	Seed size	Jiang et al. [47]
IL105, IL136	B7 _49573078	AT4G34131	UGT73B3	UDP-GlucosylTransferase 73B3	Oil content (%)	Kumar et al. [52]
IL136	A3 _20982658	AT2G15480	UGT73B5	UDP-GlucosylTransferase 73B5		

IL136	A8 _23348270	AT1G28520	VOZ1	Vascular Plant One Zinc Finger Protein	Seed yield, thousand seed weight (g), siliqua length (cm), seeds per siliqua	
IL136	A9 _35329789	AT5G41240	GSTT2	Glutathione S-Transferase THETA 2		
IL136	B2 _24459893	AT5G41220	GSTT3	Glutathione S-Transferase THETA 3		
IL136	A2 _6646330	AT3G24540	PERK3	Proline-Rich Extensin-Like Receptor Kinase 3	Thousand seed weight (g)	Mathur et al. [50]
IL136	A9 _8682715	AT1G62760	AtPMEI10	Pectin Methylesterase 10		
IL136	B3 _13463145	AT2G39700	ATEXPA4	Expansin A4		
IL136	B7 _53422098	AT4G26640	AtWRKY20	WRKY family transcriptions factors		
IL156	B5 _50510446	AT3G14310	OZS2, ATPME3	Pectin Methylesterase 3, Overly Zinc Sensitive 2		

D31_ILs = *B. carinata* derived *B. juncea* introgression lines in the genetic background of cultivar DRMRIJ 31
