# Supplementary material for: Introgression of Heterotic Genomic Segments from Brassica carinata into Brassica juncea for Enhancing Productivity
Source: Plants (Basel). 2023 Apr 17;12(8):1677. doi: 10.3390/plants12081677 (PMC10146992; doi:10.3390/plants12081677)
Supplement: Supplementary file 1 [file plants-12-01677-s001.zip › Table S9.pdf]

Table S9. Correspondence of identified candidate genes in heterotic genomic segments transferred from *B. carinata* (BC-4) into *B. juncea* cultivars Pusa Mustard 30 with already reported loci

PM30_ILs	SNP ID	*A. thaliana* ortholog	Gene name	Function	Already known to influence trait(s)	References
IL168	A1 _7414870	AT4G23060	IQD22	IQ-Domain 22	Yield related traits	Aakanksha et al. [43]
IL168, IL180, IL190	A4 _14198854	AT2G24790	BBX4, ATCOL3	B-Box Domain Protein 4, CONSTANS-Like 3		
IL168, IL187	A5 _24307089	AT1G73730	AtEIL3	ETHYLENE-INSENSITIVE3-Like 3		
IL168, IL185, IL187, IL190	A7 _26240264	AT1G74120	mTERF15	Mitochondrial Transcription Termination Factor 15		
IL168	B1 _11375013	AT5G63610	CDKE 1	Cyclin-Dependent Kinase E;1		
IL168, IL187	B3 _11420202	AT2G35940	EDA29, BLH1	BEL1-Like Homeodomain 1, Embryo Sac Development Arrest 29		
IL168	B3 _15825638	AT2G35700	ERF38	ERF family protein 38		
IL168	B4 _62883530	AT2G44940	ERF34	Ethylene-Responsive Transcription Factor 34		
IL168, IL180, IL185, IL190	B7 _51168369	AT1G28370	ERF11	ERF domain protein 11		
IL168, IL180, IL185, IL187, IL190	A7 _26240266	AT4G38160	PDE191, mTERF6	Pigment Defective 191, Mitochondrial Transcription Termination Factor 6		
IL185	B2 _45604564	AT3G47600	MYB94	MYB Domain Protein 94		
IL185	B3 _5096242	AT5G19450	CDPK19	Calcium-Dependent Protein Kinase 19		
IL187	B3 _5939846	AT5G60740	ABCG28	ATP-Binding Cassette G28		
IL187	B3 _16282672	AT1G01680	ATPUB54	Plant U-Box 54		
IL187	B3 _29815531	AT4G05460	AtSKIP19	SKP1/ASK-Interacting Protein 19		
IL187	B3 _61987951	AT3G54430	SRS6	SHI-Related Sequence 6		
IL187	B3 _62254479	AT3G54850	PUB14	Plant U-Box 14		
IL190	A4 _22172059	AT2G44940	ERF34	Ethylene-Responsive Transcription Factor 34		
IL180	B8 _1578119	AT4G33430	BAK1	BRI1-Associated Receptor Kinase	Seed size	Jiang et al. [47]
IL185	B2 _36130560	AT2G13790	BKK1, BAK7	BRI1- associated kinase 7, BAK1-LIKE 1		
IL168	A1 _7869145	AT2G40540	ATKT2	Potassium Transporter 2	Oil content (%)	Kumar et al. [52]
IL168, IL180	A9 _8798604	AT1G62640	KAS III	3-Ketoacyl-Acyl Carrier Protein Synthase III		
IL168. IL185	B7 _49573116	AT4G34131	UGT73B3	UDP-Glucosyl Transferase 73B3		
IL168	B2 _5491954	AT4G27140	SESA1, AT2S1	Seed Storage Albumin 1		
IL180, IL185	A3 _22305061	AT3G50740	UGT72E1	UDP-Glucosyl Transferase 72E1		
IL185, IL187, IL190	B3 _4746117	AT4G27140	SESA1	Seed Storage Albumin 1		
IL168	B4 _61959971	AT2G39720	BTL11, RHC2A	Zinc Finger ATL 11, RING-H2 Finger C2A	Seed yield, thousand weight (g), siliqua length (cm), seeds per siliqua	
IL168, IL180, IL185, IL190	B7 _51232110	AT1G28520	VOZ1	Vascular Plant One Zinc Finger Protein		
IL168, IL180	A9 _11281228	AT1G55050	SANT4	SANT domain		
IL185	B6 _9676843	AT1G55050	SANT4	SANT domain		
IL190	A1 _37265001	AT3G14660	CYP72A13	Cytochrome P450, Family 72, Subfamily A, Polypeptide 13		
IL190	B8 _67714450	AT1G14687	AtHB32, ZHD14	Homeobox Protein 32, zinc finger homeodomain 14		

IL168	B1 _13486179	AT5G49180	PME58	Pectin Methylesterase 58	Thousand seed weight (g)	Mathur et al. [50]
IL168	B4 _62813797	AT4G00190	ATPME38	*A. thaliana* Pectin Methylesterase 38		
IL168	B4 _62813818	AT2G45220	PME17	Pectin Methylesterase 17		
IL187	B4 _13959821	AT1G06070	bZIP69	Basic Leucine-Zipper 69		
IL168	B6 _9965964	AT1G58100	TCP8, AtTCP8	TCP Domain Protein 8	Yield related traits, thousand seed weight (g)	Aakanksha et al. [43]; Mathur et al. [50]
IL185	B6 _16324111	AT1G09770	ATMYBCDC5	*Arabidopsis thaliana* Cell Division Cycle 5		
IL168, IL180, IL185, IL187	B4 _59384705	AT3G49600	ATUBP26	Ubiquitin-Specific Protease 26	Yield related traits	Miller et al. [54]
IL187	B3 _63330777	AT3G56860	UBA2A	UBP1-Associated Protein 2A		
IL187	B3 _5578512	AT2G40930	ATUBP5	Ubiquitin-Specific Protease 5	Yield related traits	Aakanksha et al. [43]; Miller et al. [54]
IL168, IL180, IL187, IL190	A4_13933042	AT2G24450	FLA3	FASCICLIN-Like Arabinogalactan Protein 3 Precursor	Siliqua length (cm)	Wang et al. [45]

PM30_ILs = *B. carinata* derived *B. juncea* introgression lines in the genetic background of cultivar Pusa Mustard 30
